# Editorial: The Consequences of COVID-19 on the Mental Well-Being of Parents, Children and Adolescents

**DOI:** 10.3389/fpsyt.2022.924599

**Published:** 2022-05-23

**Authors:** Emma Sorbring, Ylva Svensson, Soly I. Erlandsson, Kirby Deater-Deckard

**Affiliations:** ^1^Department of Social and Behavioural Studies, University West, Trollhättan, Sweden; ^2^Psychological and Brain Sciences, University of Massachusetts Amherst, Amherst, MA, United States

**Keywords:** COVID-19, pandemic, parents, children, adolescence

The COVID-19 outbreak paralyzed the whole world. The direct and indirect effects of the pandemic range from an adverse effect on the health of individuals to financial devastation on both personal and societal levels. Early in the pandemic, it looked like children and youths were less likely to become infected, yet they were affected by extreme and sustained shifts toward social and educational distancing. On top of that, many have seen their lives drastically changed due to parents' loss of work and income. Parents might feel worried about contracting COVID-19, losing their job, or not being able to keep up with the family routines, whereas children can be sensitive to abrupt changes in the daily communication within the family and react differently to these changes.

The goal of this edition of Research Topic is to highlight the effect that closure of educational institutions and organized leisure activities, as well as parents' changed working conditions and other changes in the life of children and youth due to the outbreak of the COVID-19, have had on children and youth, parents, and family life in general. Furthermore, the aim is to illustrate the diverse ways in which the different parties recognize their own resources and to examine if and how they can adapted to the uncertainty of the situation. Finally, we want to highlight the impact on individuals as well as personal considerations about the role of the family and society.

These aims are addressed in a total of 33 studies, divided into five themes: (1) variations due to demographics and other factors, (2) pre-pandemic health and disorders, (3) impact on children's, adolescents' and parents' mental health, (4) parenting stress, lack of resources and particular circumstances, (5) support and interventions at the institutional and individual level. This Research Topic illustrates not only how fast and efficiently societies, health care services, families and youth rose to the challenges of the pandemic, but also how researchers all over the world did the same. The breadth of research questions addressed, and the diversity of disciplines show the wide impact of the pandemic. Even though it is evident from the combined results of the studies in the current edition of the Research Topic that the pandemic has had severe consequences for the mental wellbeing of parents, children, and adolescents, they also illustrate the resilience and resources of the same.

## Variation Due Gender, SES, Social Isolation, and Other Factors

Worldwide, we have met a wide array of challenges imposed by the COVID-19 pandemic, such as the impact of social isolation on health and wellbeing at different stages of young peoples' lives. Corresponding to these conditions, Prowse et al. conducted an online study among undergraduate students aiming to explore the impact of COVID-19 on school performance, social isolation, mental health, and coping strategies. Frequent use of social media indicated negative mental health effects for both male and female students. Furthermore, use of cannabis was associated with negative effects on academic outcomes in males.

In India, young people's (12–18) top worries during the pandemic lockdown were academic achievement, social and recreational activities, and physical health (Shukla et al.). Females' worries concerned academic achievement and physical health, while males worried about social and recreational activities. The significant negative impact of the pandemic on Indian adolescents calls for a need to ensure access to digital education and medical care.

The pandemic lockdowns have led to a special focus on mental health issues where adolescents may have been among the most affected (Myhr et al.). A cross-sectional Norwegian dataset from 2014 was compared to potential changes in adolescents' self-reported mental health across sociodemographic groups during lockdown in the spring of 2020. Potential changes in mental health problems and life satisfaction with reference to lock-down and socioeconomic groups were analyzed using logistical regression models. The least privileged socioeconomic groups exhibited notable psychological distress, but there was no substantial change overall? During the first wave of the pandemic.

In a cross-sectional analysis, Ramirez et al. studied mental health problems among 979 children aged 4–18 years old. The results showed that positive educational experiences, praying, and meditation reduced the probability of mental health problems, while having family or health problems increased emotional problems. The latter was true for adolescents but could not be found for children.

In February 2020, Jing et al. collected data (17,876 valid questionnaires) on self-rated symptoms of depression among Chinese University and College students. Social demographic features were gender, ethnicity, personality, residence, and educational level. Findings revealed for instance that students who were highly impacted by the pandemic outbreak had higher self-rated depressive symptoms. Furthermore, introverted students were likely to report more severe symptoms of depression.

Chai et al. performed a meta-analysis to confirm the prevalence of mental health symptoms for Chinese children and adolescents during the COVID-19 lock-down. A total of 12 studies were included and results of the meta-analysis indicated that there was an increasing number of children and adolescents who experienced mental health problems during the lock-down. It was pointed out by the authors that implementations for mental health management, especially for girls, need to be prepared.

## Pre-Pandemic Health and Disorders

Several national investigations [e.g., (1)] have called for attention when it comes to children, young people, and families that even before the pandemic, for whatever reason, were in a vulnerable situation. In this Research Topic several researchers have highlighted how children and young people's pre-existing mental and social health was associated with coping and wellbeing during the pandemic. Furthermore, authors in this Research Topic have examined pandemic lockdowns and behaviors in relation to children with diagnoses like OCD, NDD, and Autism. For example, Suzuki and Hiratani explore associations between children's activities, caregivers preventive behavior and children's and caregivers' mental health problems during the pandemic. The researcher found that caregivers' worrying about children's activities was positively associated with both their own, as well as their children's fear of the virus, and with the children's depressive symptoms.

In a review of all studies published during 2020 concerning the impact of the pandemic on mental health in adults, adolescents, and children with Obsessive Compulsive Disorder (OCD), 14 studies were selected. In an analysis of these studies, Zaccari et al. revealed that the pandemic had an impact on OCD in all age groups. Only one of the 14 studies showed a slight reduction of symptoms, while the other studies showed an increase in symptoms. The few studies about adolescents and children showed exacerbation of OCD, even in the presence of an ongoing treatment.

In a third study, 72 families with children on the autism spectrum condition (AUC), as well as 62 families without children on the AUC, were included. Fong et al., found that the families of children in the age-span 5 to 17-years-old were equally negatively affected by the lockdowns, and that families with children on the AUC were either more or less, unaffected.

The above result could also be seen in another study that compared at-risk children to the general population. Bussiéres et al., conducted a systematic review and meta-analysis including 28 empirical studies on the mental health of children aged 5–13 years, both with and without neurodevelopmental issues or chronic health issues. The result indicated no differences between children from the general population and children with pre-existing mental health problems. For both groups, children's mental health was generally negatively impacted during the pandemic.

Zijlmans et al. did a study that included 8- to 18-year-old children and adolescents that were divided into a psychiatric sample of 249 participants, a pediatric sample of 90 participants, and a general population sample of 844 participants. The psychiatric sample reported significantly more problems, such as depression, global health, and anger, than both the other groups, except for anxiety and peer relations. However, having a COVID infected friend or relative, as well as having experienced changes in parental work due to the pandemic, negatively moderated the outcome for all, except for participants with pre-existing problems.

Looking at adolescents, the patterns are in the same direction. In a sample with 24 adolescents (9 with and 15 without Early Life Stress, ELS), Cohen et al. compared symptoms of depression and anxiety before and during the pandemic. The results showed a large increase in both depression and anxiety for adolescents who, prior the pandemic were healthy, and stable, not increasing, levels of depression and anxiety during the pandemic, for adolescents experienced ELS before the pandemic.

In a study of 1,427 older young people, undergraduate students, Biondi et al. investigated the association between personal traits and compliance with pandemic behavioral recommendations. They found that students with immature defense mechanisms, as well as internalizing personality traits were at a higher risk for stress symptoms, which in turn was related to less compliance with behavioral recommendations.

## Impact on Children's, Adolescents' and Parents' Mental Health

The long-term consequences for both children and parents after months of social isolation are still unknown and follow-up studies to prevent further health risks are needed. Fasano et al. explored the impact of lockdown in an online survey that included 814 parents with children (ranging in age from 4 to 11 years). Changes in emotional state, altered routines, and sleep disorders were present in the children and there was a strong correlation between children's and parents' emotional conditions and lifestyle during lockdown. Most worried as a consequence of lockdown were families in the lower range of socio-economic status.

Sleep patterns have also been studied by Lokhandwala et al. using a within-subjects design and actigraphy-measured sleep from 16 preschool children. The results showed that children that woke up earlier had more negative expression, both before and during the pandemic. During the pandemic, those children who? Engaged in at-home learning, slept longer which in turn was associated with less negative expression. This research calls for attention to children's sleep/wake onset and coping strategies during stressful events.

Living in areas stricken by different forms of threat can dilute Posttraumatic Stress Symptom (PTSS). A survey performed in Israel by Levavi et al. explored the adverse effects of COVID-19 on mothers and their children while living in areas with high (*n* = 40) and low (*n* = 78) exposure to armed conflict. Data collection took place before and after the outbreak of the pandemic. Interview data, after the first lockdown, revealed no difference in perceived adverse effects of COVID-19 between the two groups. However, maternal PTSS and the child's efforts to be in control predicted negative effects of COVID-19, but only in the high-exposure group.

In another part of the world a three-generation cohort of? Family studies were the starting point for a COVID-19 survey during the height of the Australian lockdowns in May–September 2020 (Biden et al.). Included were 502 parents of 871 children who had completed an inventory of social support during young adulthood (2006) and in a postpartum period (2010). Pre-pandemic support from family and friends during lockdowns was positively associated with the experience of support within families but also within the local community.

The impact of the COVID-19 pandemic on children and adolescents' (0–18 years) mental health and psychiatric conditions was the focus of a literature review by Marchi et al. Due to the methodological heterogeneity of studies included, conclusions regarding the effects of COVID-19 on psychological health were somewhat complicated. Interventions such as physical activity and reduced screen time for children and adolescents, as well as support programs for parents, were recommended.

Khoury et al. accomplished a longitudinal investigation of children's internalizing and externalizing behavior in associations with parents' mental health before and during the COVID-19 pandemic. Considering child gender and COVID-related stressors, hostility in parents was associated with greater changes in externalizing problems, while maternal anxiety was associated with greater increases in internalizing problems.

## Parenting Stress, Lack of Resources and Particular Circumstances

A key aspect of the pandemic is whether and how the risks and challenges associated with infection, illness, mortality and public health measures (particularly shutting down of workplaces, schools, and shopping areas) impacted parental and family stress and mental health. Adams et al. sought to answer this question in a longitudinal online survey with a socioeconomically diverse sample of over 400 parents of children or adolescents across the United States. Two clear patterns emerged—that parenting stress climbed markedly across the beginning of the pandemic, and that 6 months into the pandemic (as children started to return to school in the fall) these stress levels had not returned to pre-pandemic levels.

Were particular factors predictive of better or worse stress and adjustment among parents? In a survey study of nearly 150 Norwegian couples with 11–13 year old adolescents, Idsoe et al. examined levels of pandemic-related trauma and stress symptoms as well as their correlates with regard to exposures and disruptions to family life during the shutdown in Norway. There was little evidence of a pandemic-era impact or effects on stress symptoms, and this was true for both women and men in the sample—perhaps due to the social safety net and less severe shutdown in Norway compared to other parts of Europe and beyond.

In stark contrast were the results reported by Whitaker et al., who conducted a survey with nearly 1,000 low-income inner London mothers and fathers of very young children in the early and later stages of pandemic-era shutdowns. They found that mental health challenges were linked with income and food insecurity, having no outdoor space for children, and lower social support. Also, symptom levels varied depending on ethnicity and parent gender, indicating the need to consider intersecting aspects of family demographic factors.

Another area of concern during the pandemic has been the impacts on women and babies during pregnancy and the neonatal period. It stands to reason that pregnant women and new mothers may be more vulnerable to pandemic-related stress, given the impacts of that stress on the developing fetus and newborn baby. In an analysis of over 200 women in northern Italy who delivered during an acute period of infection levels, Grumi et al. found links between greater emotional distress, lower social support from family and friends, and more anxious and depressive symptoms. Importantly, although overall levels of symptoms were higher than would be expected based on prior literature (suggesting a potential pandemic-era increase in symptoms overall), individual differences in exposure were unrelated to symptoms.

In another cross-sectional study of over 2,000 pregnant women in Guangzhou, China, Zheng et al. found that that maternal stressors and mood disturbance symptoms were lower prior to the pandemic, depression was highest in the first trimester and insomnia and stress symptoms in the third trimester. Additional results pointed to the importance of earlier mental health challenges as predicting more symptoms during pregnancy, and family social and instrumental support as reducing risk.

An additional aspect of pandemic-era concern for pregnant women involves whether and how their occupations during pregnancy might increase risk or buffer them from stress. In a study of over 200 pregnant women in Chongqing, China, Liu et al. investigated whether being pregnant while also being a healthcare worker during the early days of the pandemic had an impact on stress and mental health challenges. The results clearly showed a markedly higher level of certain symptoms for healthcare workers during pregnancy (including somatic problems, anxiety, and hostility), suggesting that the already typically stressful period of a pregnancy was made more so if pregnant women had to work in healthcare settings where risk of exposure was high.

Does having children (i.e., being a parent) even matter with respect to pandemic impacts on adult stress? In two survey studies conducted in the state of Washington, United States, Avery et al. examined whether and how having children in the household was related to stress and mental health among adult women in the early days of the pandemic. The first study showed that having children at home was unrelated to stress but was related to higher anxiety symptoms; the second study, utilizing a behavioral genetic twin design, found that the effect of having children on maternal stress and anxiety may be due to confounding genetic and non-genetic factors. The study provides further evidence that parents (compared to non-parenting adults) may have particular vulnerability during pandemics.

In moving forward, it is imperative that researchers and practitioners have reliable and valid measurement tools to improve assessment of pandemic-related impacts on families with children and adolescents. To that end, in their four-country measurement development and validation study, Prime et al. published the COVID-19 Family Stressor Scale. The instrument includes three scales pertaining to stress arising from income insecurity, family stress, and chaos arising from the pandemic. Between-family differences in these scales showed expected associations with parent and child mood disturbance symptoms and family problems.

## Support and Interventions at the Institutional and Individual Level

The COVID-19 pandemic with physical distancing, lockdown, and social isolation posed new challenges to mental health. This called for the allocation of resources and support at all levels, and the development of innovative interventions, like online solutions. For example, to continue to provide health care during the pandemic, online support programs were developed. Szlamka et al. evaluated a brief Hungarian mental health crisis intervention for COVID-19-related stress and challenges. The counselors observed three key features of the online delivery of the program: (1) an explicit problem-oriented approach to counseling; (2) challenges of building rapport online; and (3) frames of online counseling.

During the pandemic, learning also moved online. To better understand how that affected youth, Li et al. examined the main and interactive effects of online learning satisfaction, COVID-19-related stressors, and coping on the adjustment of Chinese secondary school pupils during the pandemic outbreak. Results showed that problem-based coping and online learning satisfaction promoted adolescents' adjustment directly or as a buffer against the negative impact of stressors on adjustment, while emotion-based coping was a risk factor, both directly and indirectly.

Despite the advantages, the increased use of technological solutions may also be associated with risks. Duan et al. found that smartphone addiction among children and adolescents in China increased during the pandemic. They developed a tree model for decision-making to be used by researchers and parents as a screening tool to assess the risk of smartphone addiction quickly and easily, based on five risk factors: (1) Internet addiction; (2) hours spent on a smartphone during the epidemic; (3) levels of clinical anxiety symptoms; (4) fear of physical injury, and (5) sex. Though professional mental health services rapidly transitioned to online delivery models, it has not been sufficient to meet the growing need. During times of quarantine and lockdown, peer support can function as a complementary resource to professional services.

A review done by Suresh et al. showed that peer-to-peer social and emotional support generally had positive effects on mental health during the pandemic. As children are returning to school after the pandemic, lessons can be learned based on previous experiences of child-focused, post-crisis interventions.

A rapid systematic review (Gómez et al.) of mental health interventions previously used to reduce mental health symptoms and sequelae among children showed that cognitive-behavioral therapy was the most common intervention type and that school-based interventions were the most common method. Finally, findings suggest that preventive programs for adolescents with pandemic-related stress should pay attention to dreams.

In a study conducted in Italy, Romania and Croatia (Guerrero-Gomez et al.) found that secondary school students reported heightened dream recall and an increase in nightmares during the lockdown. Moreover, 15% of the dreams included pandemic-related content. Further, subjective emotional reactions to lockdown had a higher correlation to dreaming than objective distress such as that caused by COVID-19-related illness or the death of someone close to them.

The pandemic situation has been hard for most people, in different ways, in different parts of the world, and in different age groups. The consequences of lockdown during the COVID-19 pandemic have been extraordinarily hard on families around the world. In this Research Topic, academics have explored a diversity of variables, situations, and conditions associated with the pandemic and contributed to extended knowledge about the consequences of the COVID-pandemic for different groups. Vulnerable groups have received attention as being extra sensitive to pandemic conditions, such as isolation and unemployment. However, this Research Topic calls for paying attention to children and youths in general and the hidden future consequences. Little is still known about the long-term effects on, for example, children's and youth's social-och cognitive development. For many young people the pandemic has meant developing habits that are not common for young people. In a period of life when adolescents strive toward greater autonomy by spending less time with parents and more time with peers (2), the demands for social isolation resulted in the opposite. Instead of spending time with peers, young people many times spent all their time with parents. The education situation changed dramatically, and all children have not had the technical or relational support that was desirable for long-term academic success. Neither school performance nor the clinical aspects of the COVID-infection have been in focus in this edition of Research Topic; however, it is critical to acknowledge the multiple changes that the pandemic brought to young people's lives and the potential long-term effects this will have for them and their families.

## Author Contributions

All authors listed have made a substantial, direct, and intellectual contribution to the work and approved it for publication.

## Conflict of Interest

The authors declare that the research was conducted in the absence of any commercial or financial relationships that could be construed as a potential conflict of interest.

## Publisher's Note

All claims expressed in this article are solely those of the authors and do not necessarily represent those of their affiliated organizations, or those of the publisher, the editors and the reviewers. Any product that may be evaluated in this article, or claim that may be made by its manufacturer, is not guaranteed or endorsed by the publisher.
